# Dioxin Toxicity *In Vivo* Results from an Increase in the Dioxin-Independent Transcriptional Activity of the Aryl Hydrocarbon Receptor

**DOI:** 10.1371/journal.pone.0015382

**Published:** 2010-11-08

**Authors:** Miguel Angel Céspedes, Maximo Ibo Galindo, Juan Pablo Couso

**Affiliations:** School of Life Sciences, University of Sussex, Brighton, East Sussex, United Kingdom; University of Louisville, United States of America

## Abstract

The Aryl hydrocarbon receptor (Ahr) is the nuclear receptor mediating the toxicity of dioxins -widespread and persistent pollutants whose toxic effects include tumor promotion, teratogenesis, wasting syndrome and chloracne. Elimination of Ahr in mice eliminates dioxin toxicity but also produces adverse effects, some seemingly unrelated to dioxin. Thus the relationship between the toxic and dioxin-independent functions of Ahr is not clear, which hampers understanding and treatment of dioxin toxicity. Here we develop a *Drosophila* model to show that dioxin actually increases the *in vivo* dioxin-independent activity of Ahr. This hyperactivation resembles the effects caused by an increase in the amount of its dimerisation partner Ahr nuclear translocator (Arnt) and entails an increased transcriptional potency of Ahr, in addition to the previously described effect on nuclear translocation. Thus the two apparently different functions of Ahr, dioxin-mediated and dioxin-independent, are in fact two different levels (hyperactivated and basal, respectively) of a single function.

## Introduction

**Figure 1:**
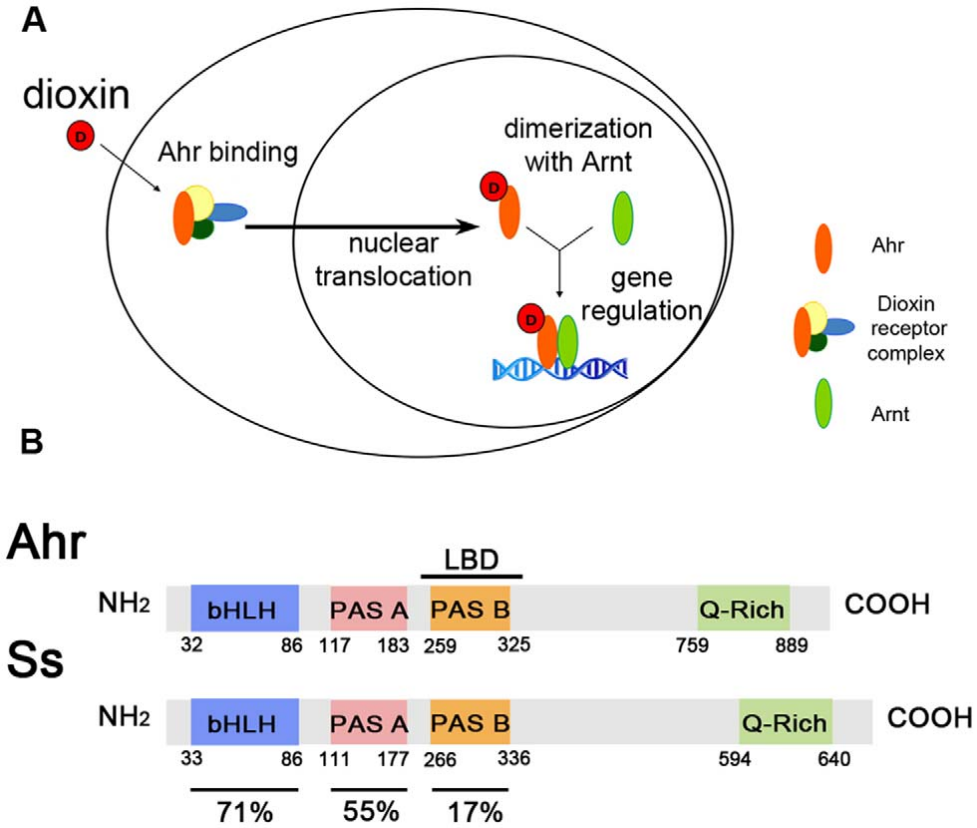
*Ahr* mediates the cellular response to dioxin. (A) Current core model of Ahr signalling in vertebrates. (B) Sequence conservation between Ahr and Ss. LBD, dioxin binding domain.

The Aryl hydrocarbon receptor (Ahr) is the key component in the metabolic response to the extremely widespread, toxic and persistent pollutants dioxins [1], [2], [3], [4]. Dioxins produce epidermal and hepatic toxicity, teratogenesis, autoimmunity and carcinogenesis [5], [6]. Ahr is a cytoplasmic bHLH-PAS transcription factor that, upon binding of dioxin, translocates to the nucleus where it forms a complex with the Aryl hydrocarbon receptor nuclear translocator (Arnt), another bHLH-PAS protein [7], and binds to an eight-nucleotide motif (Xenobiotic Response Element; XRE) to control the expression of specific target genes [8], [9] (Figure 1A). Previous work with *Ahr* knock-out mice revealed the existence of dioxin-independent functions of Ahr, including developmental functions and ageing-related detoxification [1], [2], [3]. However, the relationship between these two functions of Ahr, toxic and dioxin-independent, remains unclear [5], [10], in particular whether dioxin toxicity entails loss or excess of the dioxin-independent Ahr function, or a new independent function. We have tackled this question, which is central to the treatment of the effects of dioxins, using a *Drosophila* model for Ahr function and dioxin toxicity. *Drosophila*, like other invertebrates, does not suffer dioxin toxicity because its Ahr homologue does not bind dioxins [11], [12], but offers an extensive suite of genetic techniques. Hence, we have introduced the mouse dioxin receptor in flies, and studied its activity *in vivo* in the presence or absence of dioxin. A similar strategy (‘humanising’ or introduction of human genes in animal model systems) has allowed the study of the molecular and genetic basis of other human medical conditions in *Drosophila* (i.e. Alzheimer's, Parkinson's and Huntington's diseases) [13].

## Results and Discussion

### *Ahr* is able to fulfil dioxin-independent functions in *Drosophila*

**Figure 2:**
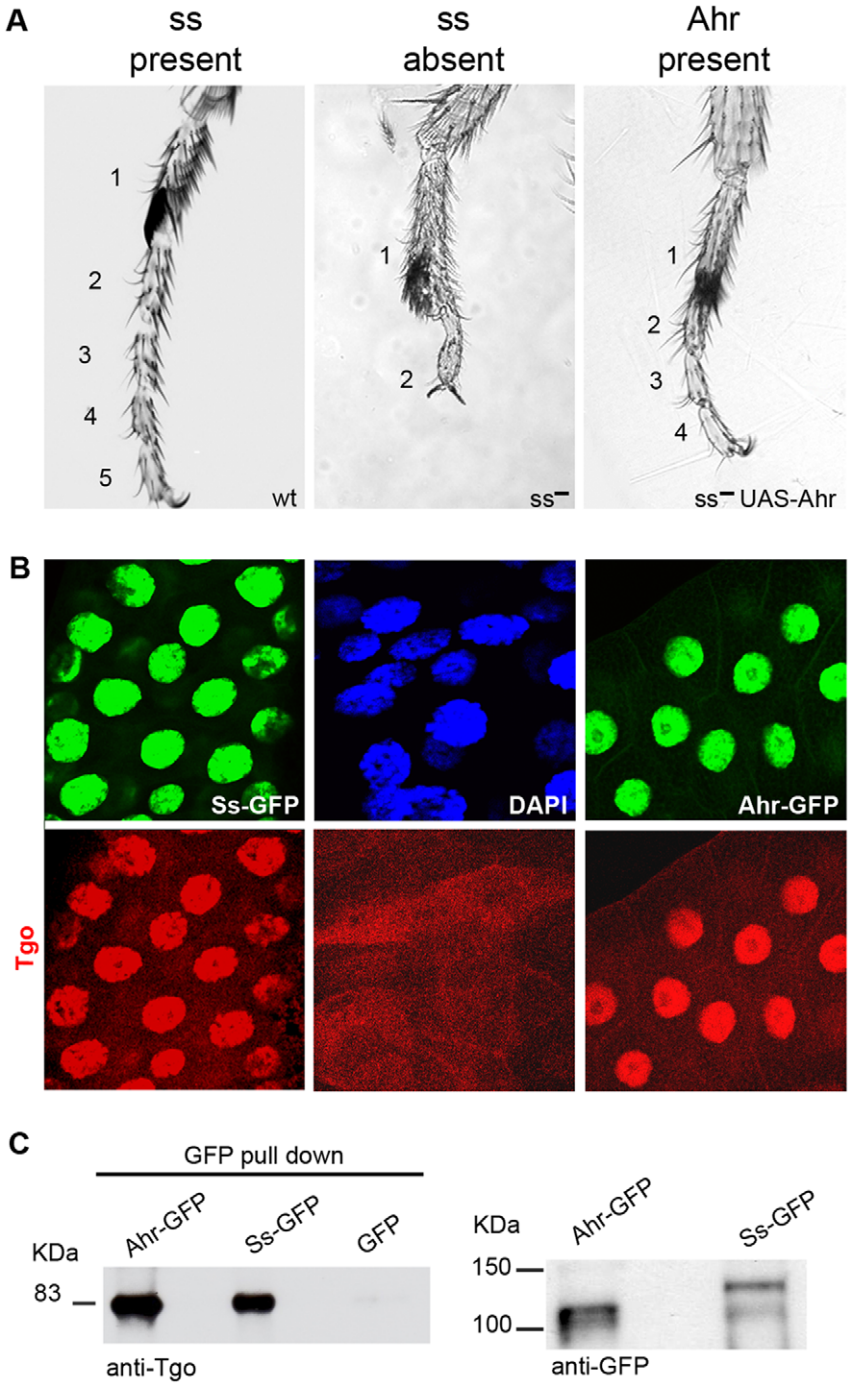
*Ahr* fulfils *ss* functions in the absence of dioxin. (A) *Drosophila* leg phenotypes; the numbers of tarsal segments present is indicated. From left to right: wild type, *ss* mutant, and *ss* mutant rescued by *Ahr* expression. (B) Larval salivary glands showing localisation of Ss-GFP and Ahr-GFP (green), Tgo (red) and DAPI (blue). Conditions as above the panels in A. Tgo is nuclear in the presence of either Ss-GFP or Ahr-GFP but not in their absence (middle panels). (C) Left, Ahr-GFP and Ss-GFP bind Tgo in *in vivo* co-immunoprecipitation assays. Ahr-GFP and Ss-GFP were precipitated using anti-GFP, Tgo was detected with anti-Tgo antibody. Right, anti-GFP reveals the presence of Ahr-GFP and Ss-GFP.

Mouse Ahr and its *Drosophila* homologous protein, Spineless (Ss), are highly similar in the bHLH and PAS A domains (Figure 1B). The *spineless* (*ss*) gene has well-characterised dioxin-independent functions during development [14], [15]. For instance, *ss* expression controls leg development by repressing the *dachshund* (*dac*) and *Bar* genes [16], [17] (Figure 2A, 3C). Ss protein locates to the nucleus [18] (Figure 2B) and its presence promotes the nuclear localisation of the fly homologue of Arnt, Tango (Tgo) which is otherwise distributed evenly across the cell [19] (Figure 2B). Ss::Tgo heterodimers target gene expression (both activation and repression) through the XRE motif both *in vitro* and during development [16], [19]. In *Drosophila*, the development of the legs and the eyes are sensitive to the precise dosage of Ss and Tgo [14], [15], [19] (see below), and thus provide an accurate read-out of ss-related activity and function.

**Figure 3:**
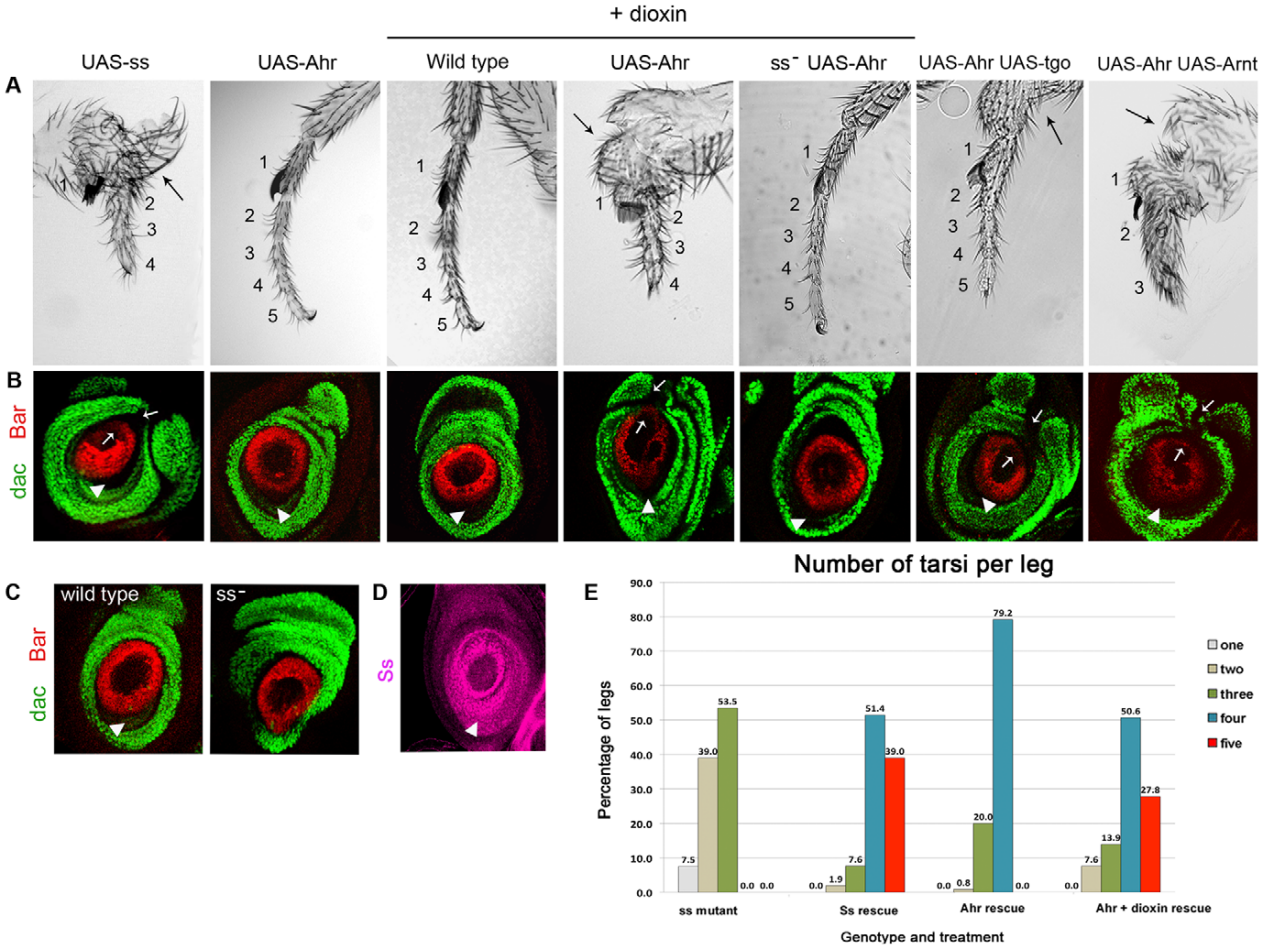
Ahr activity is enhanced by dioxin or addition of Arnt or extra Tgo. (A) Leg phenotypes. Genotypes and presence of dioxin are indicated above the panels. The number of tarsal segments present is indicated. Arrows point to proximal leg deformities. (B) Late third instar leg imaginal discs showing Dac (green) and Bar (red) expression; genotypes as above. The normal (arrowheads) and ectopic (arrows) repression of Dac and Bar is indicated. (C) Late third larval-instar leg imaginal discs showing Dac and Bar expression in a wild-type and *ss* mutant. (D) Wild-type Ss expression in leg imaginal disc during third larval instar. Notice the correlation between *ss* expression and repression of Dac and Bar (arrowheads). (E) Quantification of legs according to the number of tarsal segments displayed. From left to right: *w*; *rn-Gal4 ss^abr^/ss^sta^* (*ss* mutant), *w*; *UAS-ss/+*; *rn-Gal4 ss^abr^/ss^sta^* (*ss* rescue), *w*; *UAS-Ahr/+*; *rn-Gal4 ss^abr^/ss^sta^* (Ahr rescue) and *w*; *UAS-Ahr/+*; *rn-Gal4 ss^abr^/ss^sta^* fed with dioxin (Ahr+dioxin rescue). Notice that presence of five tarsal segments (red column) indicates full rescue of the mutant phenotype. A minimum of 80 legs, from at least 20 flies, were observed per sample (see Table S1).

Ss is not able to bind dioxins [11] probably because its PAS-B domain, which contains the dioxin binding domain [20], is highly divergent from vertebrates (Figure 1B). For this reason, we introduced mouse *Ahr* under an inducible promoter (*UAS-Ahr* or a tagged *UAS-Ahr-GFP*) in transgenic flies, and expressed it in the domain of *ss* using the *rnGal4* driver, which mimics *ss* expression [17], [21]. Once in flies, and in the absence of dioxin, Ahr is able to fulfil the dioxin-independent functions of Ss. Thus Ahr: 1) rescues almost completely the loss of the *ss* gene during development (Figure 2A, 3E); 2) localises to the nucleus (Figure 2B); and 3) binds Tgo and promotes its nuclear localisation (Figure 2B and C). We conclude that the dioxin-independent activities of Ahr and Ss are functionally equivalent, and mediated through binding to Tgo/Arnt.

### Fruit flies expressing the murine Ahr respond to dioxins

**Figure 4:**
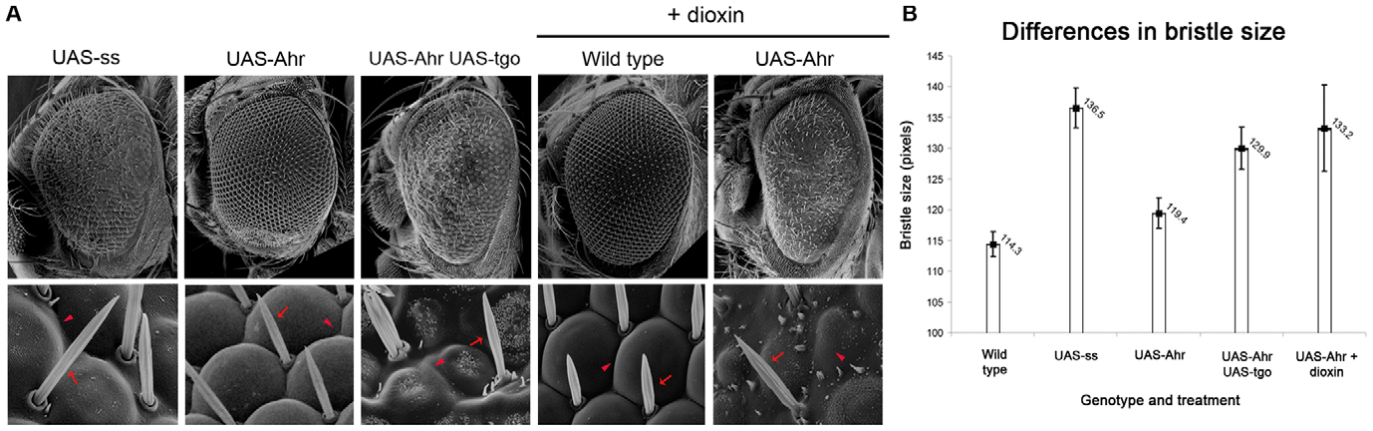
Hyperactivated Ahr produces abnormal eye development. (A) SEM pictures of adult eyes taken at 500× (top panels) and 10000× (bottom panels). Genotypes and treatment are indicated above. Notice that UAS-ss, UAS-Ahr+Tgo and UAS-Ahr+dioxin produce fused ommatidia (red arrowheads) and enlarged bristles (red arrows). Compare with the wild-type morphology (Figure S1B) shown by UAS-Ahr, and the Wild-type exposed to dioxin. These phenotypes resemble those caused by mutations in tumour-supressor genes [28]. (B) Eye bristle size average and standard errors. The genotypes are: Oregon-R (wild-type), *w*; *GMR-Gal4/+*; *UAS-ss/+* (UAS-Ss), *w*; *GMR-Gal4/UAS-Ahr* (UAS-Ahr), *w*; *GMR-Gal4/UAS-Ahr UAS-tgo* (UAS-Ahr UAS-tgo) and *w*; *GMR-Gal4/UAS-Ahr* fed with dioxin (UAS-Ahr+dioxin).

We next explored the effects of adding dioxin on the activity of both Ss and Ahr, in order to discriminate whether dioxin induces *in vivo* either a reduction or an increase in the activity of Ahr. Exposure of wild-type flies to dioxin produces no effects (Figure 3A), as expected from the inability of Ss to bind dioxin [11], [19]. However, an excess of *ss* function can be generated in the absence of dioxin by ectopic overexpression of the *UAS-ss* transgene with *Gal4* drivers. This ectopic overexpression interferes with normal development and produces leg deformities, tarsal segment loss and roughened eyes (Figure 3A, 4A), and ectopic repression of *ss*'s developmental targets *Bar* and *dac* [16], [17] (Figure 3B, C). Altogether these results show that the Ss protein acts both endogenously and ectopically in a dioxin-independent manner. In contrast, dioxin produces a marked increase on the activity of Ahr. Ectopic overexpression of *UAS-Ahr* or *UAS-Ahr-GFP* (both driven across the leg imaginal disc by *dpp-Gal4*) produces no obvious effect on either fly morphology or expression of *Bar* and *dac* (Figure 3A, B). However, when these flies overexpressing *UAS-Ahr* are exposed to dioxin, the effects mimic those obtained with *UAS-ss*: abnormal legs and eyes, and ectopic repression of *dac* and *Bar* (Figure 3A–D, 4A–B). Moreover, the presence of dioxin also allows more complete rescue of the loss of *ss* by *Ahr* than in its absence (Figure 2A, 3A, 3E, Table S1), a rescue now comparable to that exerted by the *UAS-ss* transgene itself. Altogether our results suggest that *in vivo* dioxin produces a hyperactivation of the Ahr protein. Thus, Ahr shows two levels of activity in the context of the whole organism: 1) basal low activity without dioxin, allowing only partial rescue of the endogenous *ss* gene function, and 2) high activity in the presence of dioxin, allowing Ahr to mimic the full effects of Ss, both endogenous and ectopic.

### Dioxin enhances the transcriptional potency of Ahr::Tgo complexes

**Figure 5:**
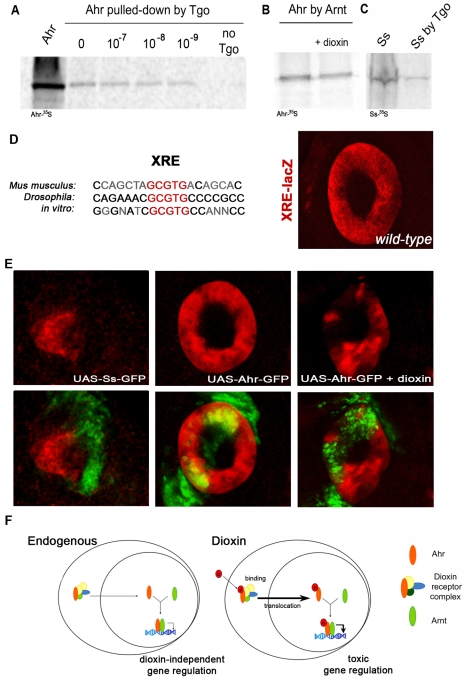
Dioxin enhances Ahr transcriptional potency. (A) Binding between Ahr and Tgo in absence or presence of dioxin in *in vitro* conditions. Ahr-^35^S is pulled-down with anti-Tgo conjugated beads. Concentrations of dioxin are indicated in nM. (B) Binding between Ahr and Arnt in absence and presence of dioxin. (C) Binding between Ss and Tgo in the absence of dioxin. (D) Left, the *Bar* XRE enhancer sequences and its conservation with the XRE from the mouse cytochrome P450 1a1 promoter and an *in vitro* probe that binds to Ahr::Arnt complexes [8] (the core XRE motif is in red and non conserved bases are in grey); right, lacZ reporter expression (red) driven by this enhancer (XRE-lacZ) in a wild-type background. (E) Repression of the XRE-lacZ reporter by Ss-GFP and Ahr-GFP in presence of dioxin, but not by Ahr-GFP alone. GFP is shown in green in the bottom panels. (F) Revised model of Ahr signalling. Our results corroborate that Ahr activity exists in the absence of dioxin and show that dioxin not only enhances the nuclear translocation of Ahr [29] but also enhances Ahr's transcriptional activity.

In vertebrates, binding of dioxin to Ahr triggers its translocation from the cytoplasm to the nucleus, followed by binding to nuclear Arnt and subsequently to XRE sequences [8] (Figure 1A). However, in *Drosophila*, the nuclear localisation of Ahr and Ss does not depend on dioxin (Figure 2B), probably due to differences in the proteins that retain Ahr in the vertebrate cytoplasm [22], [23]. Thus, our system suggests that nuclear translocation is not the only limiting step in Ahr function and that the observed hyperactivation of Ahr by dioxin must rely on another mechanism. In genetic experiments, the function of Ss is very sensitive to the dosage of Tgo. Removal of a copy of *tgo* enhances the loss of *ss* [19] and, reciprocally, the phenotype caused by ectopic Ss in the eyes can be suppressed almost completely by reducing the dosage of Tgo (unpublished observations). Correspondingly, an increase of Tgo enhances the function of Ahr, in fact, increasing the dosage of Tgo or Arnt mimics Ahr's hyperactivation by dioxin. Thus, flies overexpressing Ahr plus Tgo or Arnt display the same phenotypes as those overexpressing either Ss, or Ahr plus dioxin (Figure 3A, 3E, 4). This result is particularly noteworthy since overexpression of Tgo, Arnt or Ahr on their own produces no discernible alteration of eye or leg development (Figure 3 and S1). The similarity between the effects of dioxin and extra amounts of Tgo or Arnt indicates that firstly, our observed hyperactivation of Ahr by dioxin is bona fide, and secondly, that the endogenous amount of Tgo protein is limiting and dioxin allows Ahr to use Tgo more efficiently in the process involved. One possibility is that dioxin increases or stabilises the Ahr::Tgo complexes, but we do not observe an increase in binding between Ahr and Tgo or Arnt in Co-immunoprecipitation experiments in the presence of dioxin (Figure 5A, B). A second possibility is that dioxin increases the potency of the Ahr::Tgo complexes, in particular their transcriptional efficiency. To test this, we used a reporter gene containing the XRE enhancer sequence from the *Bar* gene that Ss::Tgo complexes bind to [16] (Figure 5D). Our results show that in the absence of dioxin Ahr is not able to repress this reporter (Figure 5E), despite forming complexes with Tgo as efficiently as Ss (Figure 1C, 5A, 5C). On the contrary, and as expected, transcriptional repression is observed in the presence of dioxin (Figure 5E).

In summary, our results show that dioxin increases *in vivo* the dioxin-independent activity of Ahr, and hence that dioxin toxicity is best understood as an excess of function of Ahr. The two postulated functions of Ahr (dioxin-dependent and dioxin-independent) in fact seem to correspond to two different levels of a single Ahr activity, basal and hyperactivated. This model envisages that hyperactivated Ahr will not only regulate more strongly the same genes as basal Ahr, but also that some genes could only be bound and regulated by hyperactive Ahr or Ss proteins [24]. In either case, in absence of dioxin, Ahr would not be able to disrupt normal development or fully rescue *ss^−^* phenotypes as Ss and hyperactive Ahr do. Furthermore, our results also suggest that the dioxin-mediated toxicity of Ahr is not solely due to inducing its nuclear translocation or to changes in affinity for its dimerisation partners, but also entails an increase of the transcriptional regulatory activity of Ahr (Figure 5F). More generally, our results corroborate that reduction, but not elimination, of endogenous Ahr and Arnt function may be the best approach to treat dioxin toxicity, and introduce *Drosophila* as a new system to study *in vivo* the genetic and molecular basis of such toxicity.

## Materials and Methods

### Reagents and fly strains

*UAS-Ahr*, *UAS-Ahr-GFP*, *UAS-ss*, *UAS-ss-GFP* and *UAS-Arnt* transgenic lines were generated by cloning the appropriate full cDNAs [14], [25] into a pUASt vector, followed by injection of purified plasmid into embryos by Vanedis and recovery of *w^+^* lines. The *ss-GFP* cDNA was generated by cloning the GFP coding sequence 3′ and in frame with the *ss* coding sequence. *rn-Gal4* [17], [21], *ss* mutants [14], and *UAS-tgo* [19] have been previously described. *dpp-Gal4*, *hs-Gal4*, *GMR-Gal4* and *AB1-Gal4* were obtained from Bloomington Stock Center.

The dioxin 2,3,5,7-tetrachlorodibenzo-p-dioxin (TCDD) was fed to parental flies and larval offspring, diluted in the food at a final concentration of 200 ngr TCDD/gr food [26]. To minimize exposure to dioxin-contaminated food and fly particles, cultures were left for nine days after first eclosions, then filled with SH medium (ethanol 100%∶glycerol, 3∶1; v/v), and the whole contents sieved for fly corpses and pupae, from which legs were dissected.

Anti-Tgo and anti-Dac were obtained from the Developmental Studies Hybridoma Bank (DSHB). Anti-Arnt, anti-GFP and anti- β-galactosidase were from Affinity BioReagents, Roche and Cappel, respectively. Anti-Bar and anti-Ss were provided by T. Kojima [16] and Yuh-Nung Jan [18], respectively. Leg discs and salivary glands were stained as described in the online methods.

### Immunohistochemistry

Third instar larvae were collected and dissected in cold PBS and fixed at RT for 10 min. in 4% paraformaldehyde. Larvae were washed in PBTx (0.03% Triton X-100 in PBS) blocked in PBTA (0.4% Bovine Albumin in PBTx). Samples were incubated overnight at 4°C in the primary antibody and in the secondary for 2hr at RT. Samples were mounted in Vectashield. Fluorescence pictures were taken in a Zeiss Axiovert confocal microscope equipped with a LSM520 Meta.

### Cuticle and eye preparations

Pharates and eclosed adults were kept in SH medium (ethanol 100%∶glycerol, 3∶1; v/v) and dissected in 70% ethanol. Samples were incubated for 5 minutes at 60°C in NaOH 1M, and then cooked in Hoyer's medium (Hoyers: lactic acid, 1∶1; v/v) for 20 min at 60°C. Finally the legs were mounted in Hoyer's medium. Adult cuticle preparations were observed under a Leica DMRB optical microscope, and the pictures were taken with one of the following softwares: Q-win or Simple PCI 6.6.

Adult flies were dehydrated by washing for 10 min in 70%, 80%, 90%, and 100% ethanol, and finally washed twice for 10 min in hexamethyldisilazane. Then samples were allowed to dry before coating them with a layer of gold/palladium alloy. Eyes were scanned in a Leo420 Steroscan SEM.

### Estimation of bristle size

SEM pictures taken at 1000× were analyzed by the imaging program Image J. Sections of the 500×200 pixels from the dorsal part of the eye were cropped. The resulting image was binarized using an appropriate threshold of brightness to isolate the bristles from the rest of the ommatidia. The background was removed by eroding once. The effect of the erosion on bristle size was corrected by dilating once. The size of each bristle was estimated by the number of covered pixels. The output data below 80 pixels or above 300 pixels were discarded to remove any artefacts caused by the background (Figure S1B). These limits were determined empirically. Between five and ten eyes were measured per sample.

### Cloning

*ss* coding sequence was extracted from *pGEX-2T-ssA1* (provided by Dr. I. Duncan) by PCR (forward primer GAGAGATCTAGAATCCGCCCTAGCAATGAGCCA, and reverse primer CCGGGAGCTGCATGTGTCAGAGG). An XbaI restriction site was inserted upstream of *ss* in a tail in the forward primer. *ss* was then sub-cloned in an XbaI restriction site in *pBK-eGFP*, leaving the *eGFP* ORF in frame with the *ss* N-terminal side. *eGFP-ss* was then cloned in the *pUASt* vector into the NotI and SpeI restriction sites.

*Ahr* and *Ahr-eGFP* coding sequences were extracted from the construct *pEGFP-Ahr* (provided by Dr. P. Fernandez-Salguero) and cloned into XbaI in *pUASt*.

*Arnt* cDNA was extracted from *pCMV-sport6-Arnt* (obtained from Gene Service), identified as clone AV-21A3. Arnt was cloned in pUASt in the EcoRI and XhoI restriction sites.

### Co-immunoprecipitation assays

For *in vivo* assays larvae *hs-Gal4 UAS-Ahr-GFP*, *hs-Gal4 UAS-ss-GFP* and *hs-Gal4 UAS-GFP* were heat shocked for 2 hours at 37°C and lysated. Mouse anti-GFP bound to Protein-G Sepharose was used to precipitate Ahr-GFP, Ss-GFP and GFP. Presence of Tgo and GFP proteins was detected by Western-blotting. For *in vitro* assays Ahr, Ss, Tgo and Arnt were expressed in rabbit reticulocyte lysates (Promega) and labelled with ^35^S as required. Binding was performed in HEPES 20 mM pH 7.9, NaCl 50 mM, 1% Np40. Anti-Tgo and anti-Arnt bound to Protein-G Sepharose were used to precipitate either Ahr-^35^S or Ss-^35^S. When described, dioxin was added during the binding reaction and washes. The range of concentrations was choosen following published data [27]. Presence of ^35^S-labelled proteins was detected with a Typhoon TRIO scanner.

## Supporting Information

Table S1 **Quantification of leg phenotypes presented in** **Fig. 3E** **.** Genotypes: ss mutant (*w*; *CyO/+*; *rn-Gal4 ss^abr^/ss* ^sta^), ss rescue (*w*; *UAS-ss/+*; *rn-Gal4 ss^abr^/ss* ^sta^), Ahr rescue (*w*; *UAS-Ahr/+*; *rn-Gal4 ss^abr^/ss* ^sta^), and Ahr + dioxin rescue (*w*; *UAS-ss/+*; *rn-Gal4 ss^abr^/ss* ^sta^+200 ngr TCDD/gr. food). Total number of legs and total number of flies (in brackets) are indicated. A number of legs is lost during dissection or due to sticking to the food or the pupal cases (see material and methods). Legs were classified according to the number of tarsi present (from one to five). The number of *ss* mutant flies is higher as they were obtained as internal control segregates in every experiment.
(DOC)

Figure S1 **Ectopic expression of either Tgo or Arnt does not affect leg development.** (A) First thoracic leg of a male (top panels) and expression of Dac (green) and Bar (red) in leg imaginal disc during third instar (bottom panels). (B) SEM pictures of adult eyes taken at 500× (top row) and 1000× (bottom row). Genotypes are indicated above panels. UAS-Tgo does not affect the wild-type eye phenotype. The binarization and measurement process is shown.
(TIF)
