# Supplementary material for: Dioxin Toxicity In Vivo Results from an Increase in the Dioxin-Independent Transcriptional Activity of the Aryl Hydrocarbon Receptor
Source: PLoS One. 2010 Nov 8;5(11):e15382. doi: 10.1371/journal.pone.0015382 (PMC2975661; doi:10.1371/journal.pone.0015382)
Supplement: Table S1 — Quantification of leg phenotypes presented in Fig. 3E . Genotypes: ss mutant (w; CyO/+; rn-Gal4 ssabr/ss sta), ss rescue (w; UAS-ss/+; rn-Gal4 ssabr/ss sta), Ahr rescue (w; UAS-Ahr/+; rn-Gal4 ssabr/ss sta), and Ahr + dioxin rescue (w; UAS-ss/+; rn-Gal4 ssabr/ss sta+200 ngr TCDD/gr. food). Total number of legs and total number of flies (in brackets) are indicated. A number of legs is lost during dissection or due to sticking to the food or the pupal cases (see material and methods). Legs were classified according to the number of tarsi present (from one to five). The number of ss mutant flies is higher as they were obtained as internal control segregates in every experiment. (DOC) [file pone.0015382.s001.doc]

**Table S1**

| **Genotype** | **n. legs (n. flies)** | **n. legs with a given number of tarsi** | | | | |
| --- | --- | --- | --- | --- | --- | --- |
|  |  | **one** | **two** | **three** | **four** | **five** |
| ss mutant | 492 (85) | 37 | 192 | 263 | - | - |
| ss rescue | 105 (27) | - | 2 | 8 | 54 | 41 |
| Ahr rescue | 120 (30) | .- | 1 | 24 | 95 | - |
| Ahr + dioxin rescue | 79 (23) | - | 6 | 11 | 40 | 22 |
