# Supplementary figures and images for: Dioxin Toxicity In Vivo Results from an Increase in the Dioxin-Independent Transcriptional Activity of the Aryl Hydrocarbon Receptor
Source: PLoS One. 2010 Nov 8;5(11):e15382. doi: 10.1371/journal.pone.0015382 (PMC2975661; doi:10.1371/journal.pone.0015382)

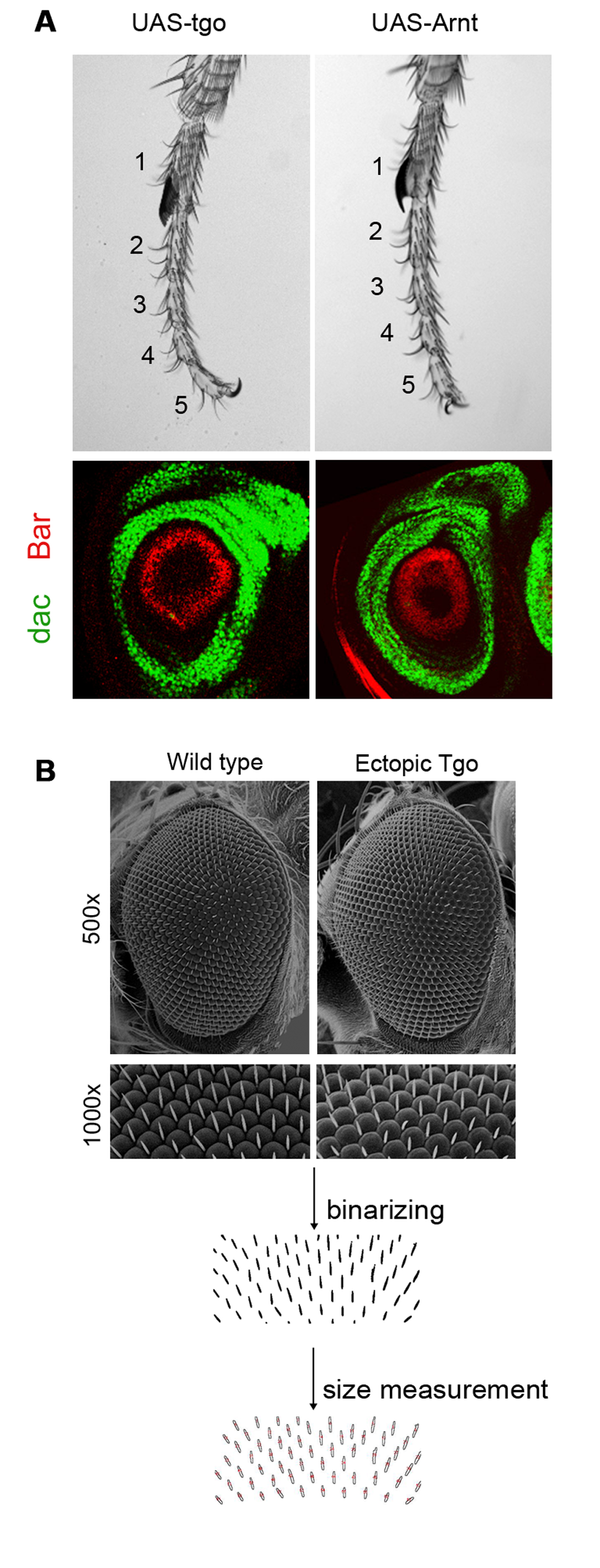

Supplement: Figure S1 — Ectopic expression of either Tgo or Arnt does not affect leg development. (A) First thoracic leg of a male (top panels) and expression of Dac (green) and Bar (red) in leg imaginal disc during third instar (bottom panels). (B) SEM pictures of adult eyes taken at 500× (top row) and 1000× (bottom row). Genotypes are indicated above panels. UAS-Tgo does not affect the wild-type eye phenotype. The binarization and measurement process is shown. (TIF) [file pone.0015382.s002.tif]
